# The influence of an acute bout of aerobic exercise on cortical contributions to motor preparation and execution

**DOI:** 10.14814/phy2.12178

**Published:** 2014-10-29

**Authors:** Jonathan S. Thacker, Laura E. Middleton, William E. McIlroy, W. Richard Staines

**Affiliations:** 1 Department of Kinesiology, University of Waterloo, Waterloo, Ontario, Canada

**Keywords:** Aerobic exercise, Bereitschaftspotential, electroencephalography, motor evoked potentials, N30, readiness potential, supplementary motor area

## Abstract

Increasing evidence supports the use of physical activity for modifying brain activity and overall neurological health. Specifically, aerobic exercise appears to have a positive effect on cognitive function, which some have suggested to be a result of increasing levels of arousal. However, the role of aerobic exercise on movement‐related cortical activity is less clear. We tested the hypothesis that (1) an acute bout of exercise modulates excitability within motor areas and (2) transient effects would be sustained as long as sympathetic drive remained elevated (indicated by heart rate). In experiment 1, participants performed unimanual self‐paced wrist extension movements before and after a 20‐min, moderate intensity aerobic exercise intervention on a recumbent cycle ergometer. After the cessation of exercise, Bereitschaftspotentials (BP), representative cortical markers for motor preparation, were recorded immediately postexercise (Post) and following a return to baseline heart rate (Post[Rest]). Electroencephalography (EEG) was used to measure the BP time‐locked to onset of muscle activity and separated into three main components: early, late and reafferent potentials. In experiment 2, two additional time points postexercise were added to the original protocol following the Post[Rest] condition. Early BP but not late BP was influenced by aerobic exercise, evidenced by an earlier onset, indicative of a regionally selective effect across BP generators. Moreover, this effect was sustained for up to an hour following exercise cessation and this effect was following a return to baseline heart rate. These data demonstrate that acute aerobic exercise may alter and possibly enhance the cortical substrates required for the preparation of movement.

## Abstract (precis)

e12178

Increasing evidence supports the use of physical activity for modifying brain activity and overall neurological health. Using electroencephalography (EEG) time‐locked to onset of muscle activity, we showed that cortical activity associated with movement preparation was influenced following aerobic exercise, evidenced by an earlier onset, and greater amplitude. These data demonstrate that acute aerobic exercise may alter and possibly enhance the cortical substrates required for the preparation of movement.

## Introduction

An increasing amount of evidence supports the use of physical activity for modifying brain activity (Hillman et al. 2008). Even single bouts of aerobic exercise have been shown to improve later sensory cortical processing across the lifespan (Hillman et al. 2003, 2009). Some attributed these modulations to changes in arousal (Kamijo et al. 2004a; Lambourne and Tomporowski 2010). The arousal hypothesis is based on findings that aerobic exercise facilitates an increase in release of brain neurotransmitters (Chaouloff 1989) resulting in an enhanced physiological readiness to respond (Sanders 1983). This is also thought to translate behaviorally to improvements in the speed of response during a choice speeded reaction task (McMorris et al. 2011). A speeded reaction task requires cortical processes including stimulus detection, stimulus evaluation, motor preparation and execution.

Current literature has primarily focused on characterizing the influence of aerobic exercise on executive control processes (Hillman et al. 2003). The event‐related potential (ERP) P300 or P3 has been employed to probe cortical processes underlying the categorization and evaluation of a stimulus (Kutas et al. 1977; Polich and Kok 1995). However, results regarding aerobic exercise on the P300 and response times contradict, with some studies displaying quicker response times and no change in P300. This discrepancy in the literature is often attributed to variations in methodology such as exercise protocol and participant characteristics (Stroth et al. 2009; Chang et al. 2012). The possibility exists that other cortical contributions such as the motor preparatory and primary motor areas could be responsible for the variations between response times and P300 morphology following aerobic exercise. Although there is an abundance of studies characterizing the P300 morphology following aerobic exercise (Hillman et al. 2003; Kamijo et al. 2004b; Audiffren et al. 2009; Stroth et al. 2009), there has been little investigation of the influence of aerobic exercise on motor‐related cortical events. Reports using the contingent negative variation (CNV), an ERP that contains both cognitive and motor generators (Loveless and Sanford 1974; Vidal et al. 1995; Brunia and van Boxtel 2001) supports an enhancement of amplitude following an acute bout of differential intensity aerobic exercise on motor preparatory cortical regions (Kamijo et al. 2004a).

Since its discovery nearly 50 years ago by Kornhuber and Deecke (Kornhuber and Deecke 1965), the internally generated Bereitschaftspotential (BP) has been used as a marker for assessing many different neurological processes including motor preparation and execution. The BP is a movement‐related potential synonymous with the readiness potential that supersedes motor production by approximately 1500 ms. The BP is commonly broken down into two main components, early and late BP. They are often distinguished by their characteristic change in slopes (occurring around 500 ms prior to movement) and named for their sequential distribution (Vaughan et al. 1968; Shibasaki and Hallett 2006; Colebatch 2007). The early BP has traditionally been associated with the activity of the supplementary motor area (SMA), and represents early motor planning and selection (Shibasaki and Hallett 2006). The late BP is associated with activity in both the SMA and the primary motor cortex and is representative of a shift from early movement preparation (SMA activity) to motor production (M1 activity) (Ikeda et al. 1992; Cui et al. 1999). Finally, the terminal region (~100 ms) of the late BP is known as the motor potential (MP) and is generated within the contralateral M1 to the moving limb. The latency and morphology of these potentials is heavily weighted on the attributes of the movement itself, with force, velocity and precision of movement influencing both BP components differently (Colebatch 2007). The current study investigated the influence of an acute bout of moderately intense aerobic exercise on the cortical networks involved in motor preparation and execution, measured by the BP.

**Figure 1.**
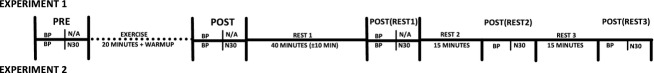
Displays the time course of events for experiment 1 and 2 protocols. The five time measures surrounding exercise are represented as Pre, Post, Post, Post(Rest1), Post(Rest2) and Post(Rest3). Each time measure is broken into BP blocks representing 8 min collection times and N30 blocks represent 5 min. Exercise time depicted by a dotted line represents 20 min of exercise plus a warm up. The Rest1 condition was on average 40 min (40.9 ± 10.4 min, *n* = 23). Experiment 2 contains both Rest2 and Rest3 which are both 15 min in duration. The primary difference between experiments 1 and 2 was the inclusion of N30 (shown in experiment 1 as not applicable [N/A]) and Post Rest2 and Post Rest3 time measures.

There were two primary objectives of this experiment. In the first experiment, we investigated whether an acute bout of moderate intensity aerobic exercise modulates cortical excitability associated with the preparation of movement. Aerobic exercise enhances autonomic sympathetic drive which centrally appears as changes in central nervous system (CNS) arousal (enhanced neurotransmitter release and readiness to respond) and peripherally as changes in heart rate. Therefore, we hypothesized that movement‐related cortical excitability would be transiently enhanced following aerobic exercise, manifested as changes to early BP onset and late BP amplitude immediately after a single session of exercise returning to pre‐exercising levels after heart rate had returned to baseline. Experiment 1 determined that acute aerobic exercise altered the morphology of early but not late BP and that this effect was maximal following the return of heart rate to baseline. Further investigation in Experiment 2 sought to characterize the transience of the aerobic exercise effect on early BP. In addition, peripheral nerve stimulation to the median nerve was used to elicit the short‐latency somatosensory evoked potential N30 in an effort to determine the generalizability of this effect within motor preparatory regions (Fig. 1). It was hypothesized that the aerobic exercise effect on early BP would progressively decay within 30 min following a return of heart rate to baseline. Moreover, we hypothesized that acute aerobic exercise would enhance N30 amplitude and that this effect would be comparable in both magnitude and transience to that of the early BP.

## Materials and Methods

### Participants

A total of 22 healthy volunteers were tested in two experiments: 11 subjects (six males, five females; average age [±SE] 27.09 years ± 4.7) participated in experiment 1 and 11 (six males, five females; average age (±SE) 24.72 ± 3.5 years) in experiment 2, with no repeated participants. Subjects were required to be eligible for exercise according to the Physical Activity Readiness Questionnaire (PAR‐Q) and able to perform an acute bout of aerobic exercise on a recumbent cycle ergometer. In addition, individuals could not be receiving treatment (and have no history of being treated) for a major neurological illness or event such as seizures/concussion. In experiment 2, participants also completed the International Physical Activities Questionnaire (IPAQ). Experiment 2 participants were characterized by a healthy body mass index range (Mean BMI: Male [26.0 ± 3.6 kg/m^2^], Female [23.5 ± 2.1 kg/m^2^]), and IPAQ classification as highly active (Mean IPAQ score: Male [4325.9], Female [4792.9]). Experimental procedures were approved by the Office of Research Ethics at the University of Waterloo. All subjects gave their informed consent to participate and were paid a nominal fee for their participation in the study.

### Exercise

Participants performed a 2 min self‐paced warm‐up followed by a 20‐min bout of exercise at 70% of age‐predicted maximum heart rate (i.e., maximum heart rate = 220 – age) on a recumbent cycle ergometer. The exercise protocol required the use of the lower limbs, with hands resting on the handle bars in an effort to remove confounding or fatiguing factors that have been previously shown to alter BP morphology (Dirnberger et al. 2004). The duration and intensity of exercise was selected to be similar to studies that have shown modulations in other neuroelectric indices (Tomporowski 2003; Hillman et al. 2009; Kamijo et al. 2009) and met the minimum criteria by the Canadian Society for Exercise Physiology in their Canadian Physical Activity Guidelines (Tremblay et al. 2011).

**Figure 2.**
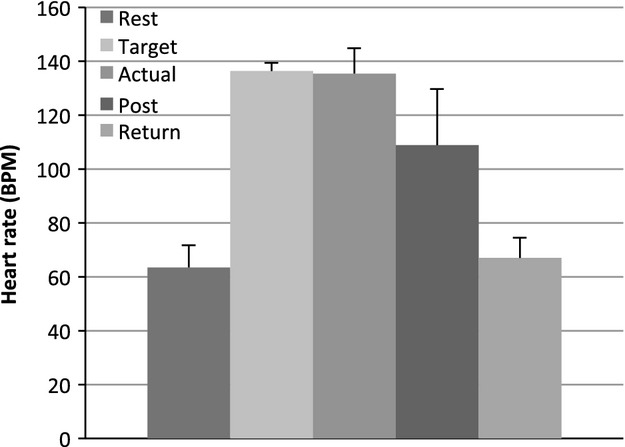
Heart rate data (*n* = 23) in beats per minute (bpm) at all time points taken with error bars depicting standard deviation. Group average heart rates at time of rest (63.5 ± 8.13 bpm), targeted exercise (136.3 ± 3.1 bpm), actual exercising (135.5 ± 9.5 bpm), immediately post exercise (108.8 ± 20 bpm), and following Rest1 heart rates (66.1 ± 7.4 bpm) are displayed. Actual exercising heart rate is the grand average of each individual's average of 5 measures taken every 4 min during exercise.

Participants resting heart rate was measured using a wristwatch‐to‐chest monitor (Polar Electro; Kempele, Finland) and taken prior to, during and immediately after exercise. Heart rate was assessed as a proxy for arousal after exercise. After completing the second motor task block (immediately after exercise), subjects were given a resting period where their heart rate was monitored continually every 3 min until it had returned to within a predetermined three beats relative to pre‐exercising values. Once subjects were deemed to have returned to a resting state, the Post(Rest) testing block commenced, on average this occurred 40.9 ± 10.5 min (Experiment 2) after exercise cessation (see Fig. 2).

In order to reduce the possibility that self‐monitoring could have on BP manipulation, the exercise intensity monitoring procedures were slightly altered between experiments 1 and 2. In experiment 1, the participant maintained their target heart rate by self‐adjusting their cadence at a constant load. In experiment 2, participants maintained constant cadence of 60 revolutions per minute and the workload was adjusted during exercise to maintain target heart rate.

### Motor task

All motor testing was completed in a sound proof booth with participants seated in a desk chair with elbow and forearm of both arms resting on a tabletop. Participants were then instructed to perform a ballistic wrist extension with their right hand, under their own pace every 3–6 sec continuously for 8 min while avoiding counting. To ensure consistency between movements, participants’ hands grasped a mobile handle that was fixed to the table and allowed for complete range of motion. Following every brisk movement, subjects were told to return to a neutral hand position before beginning the next movement.

### Transcutaneous electrical nerve stimulation

In addition to the experiment 1 protocol, experiment 2 also involved electrical stimulation of the median nerve at the right wrist in order to evoke the N30 somatosensory evoked potential. The nerve electrodes were placed on the anterior surface of the wrist approximately 2 cm laterally from the midline and then readjusted until a visible thumb twitch was detected during initial EEG and EMG set‐up. Threshold voltages were detected manually by slowly increasing stimulus voltage until a visible motor twitch was elicited within the thumb. Threshold was determined as the lowest voltage that produced a muscle twitch. Testing stimuli consisted of 1.2× the determined threshold stimulus. The stimulus characteristics consisted of a 0.5 ms square wave electrical pulse, with a 0.75 sec inter stimulus interval. Stimulation pulses were delivered using a stimulator (Grass SD‐9, West Warwick, RI) controlled by a custom National Instruments LabVIEW™ (Austin, TX) program. Following each movement task testing block, participants were instructed to keep their hand strapped within the apparatus, resting at the neutral position for the duration of the stimulation block. Each median nerve stimulation block lasted 300 sec and consisted of 120 stimulations; providing an adequate amount of data for artifact‐free single trial averaging. In total, five blocks of median nerve stimulation were conducted following each BP test block. N30 ERP data were produced by separating epochs of the continuous EEG profile between −100 ms prior to and 500 ms post stimulation.

### EMG and EEG recording procedures

Surface electromyography was recorded from the right extensor carpi radialis (ECR) muscle using bipolar electrodes placed in close proximity and oriented longitudinally over the muscular belly in both experiments. Before application of the electrodes the skin was first abraded using an exfoliating gel and rubbing alcohol. EMG was continuously collected at an amplification of 1000× and digitized at sampling rates coinciding to the sample rates of EEG data.

Scalp EEG was recorded from nine channels (FC3, FCz, FC4, C3, Cz, C4, CAP, CPz, CP4) as well as electrooculography (EOG) at the frontal orbital (EOGu, EOGl) sites for measurement of blink artifact. These electrodes were placed using a cap containing 64 electrode sites in accordance with the international 10–20 system (Quick‐Cap, Neuroscan, Compumedics, Charlotte, NC). The skin above and below the left eye, and behind the ears was cleaned with exfoliating gel and rubbing alcohol to place electrodes for EOG and mastoid references, respectively. The electrode cap was actively referenced for collection and then rereferenced to linked mastoid electrodes during analysis. All electrodes (EOG, EEG and mastoids) were filled with conductive gel and all channels had impedances of 5 kΩ or less. EEG was amplified 20,000× and digitized at a sampling rate of 500 Hz for experiment 1 and 1000 Hz (Synamps2, NeuroScan 4.5, Compumedics) for experiment 2 before exporting for post‐analysis. All post processing of data was done using either Neuroscan© (Compumedics) or LabVIEW (National Instruments Corporation).

### Data analysis

#### Movement‐related cortical potentials

EEG epochs were extracted to onset of muscular contraction as determined visually by a definitive increase in slope followed by muscular burst in EMG recordings. Epoch intervals included −2000 ms prior and 500 ms post muscle onset. Epochs with contamination (i.e., blink or movement artifact) were identified through visual inspection and removed. Individual artifact‐free epoch data were averaged (~100 epochs/participant/time point) for each subject and group averaged within each condition. Individual BP averages were resolved into four separate components; of primary interest were the early and late BPs while secondary measures included motor potential and reafferent potentials (RAP), all of which were recorded from fronto‐central (FCz) and central electrode sites (Cz). Early BP onset required a low‐pass filter of 5 Hz conducted on individual BP averages. The first 200 ms of the epoch was averaged and used as a baseline. BP onset was determined as the point at which the negativity exceeded 2 standard deviations from the baseline criterion. In order to evaluate Late BP slope, data were band pass filtered between 0.1–30 Hz. Late BP slope was determined by evaluating the slope of the EEG tracing for a time window of 500 ms prior to EMG onset. Using the same filtered data, motor potential was established by evaluating the peak to peak difference between the absolute negative peak (time window: −50 to 150 ms) and the amplitude at 150 ms prior. Finally reafferent potential amplitude was determined to be the peak to peak difference between the absolute negative peak (time window: −50 to 150 ms) and the greatest positive amplitude in the 400 ms following the absolute negative peak. Analysis was conducted using either a custom Neuroscan© (Compumedics) and LabVIEW^™^ (National Instruments Corporation) program.

#### Short latency somatosensory evoked potentials

Frontal N30 data were partitioned into epochs and time‐locked to the median nerve stimuli. Epoch intervals consisted of −100 ms prestimulus baseline followed by 500 ms post stimulus onset and were extracted from Cz and FCz electrode sites. Individual epochs were visually inspected and those containing blink artifacts were removed. Artifact‐free data were then averaged (~100 epochs/participant/timepoint) and N30 peak data analysis was conducted using two different methods. Peak N30 was defined as the most negative amplitude during a time window between 25 and 35 ms in relation to a prestimulation baseline. N30 amplitude was determined as the peak–peak difference between the N30 (25–35 ms) and the preceding positive P20 (18–22 ms) component.

#### Electromyography

Raw EMG data were separated into epochs from 100 ms prior to visually detected onset to 500 ms post onset, band‐pass filtered between 20 and 500 Hz (to remove nonbiological noise), baseline‐corrected and full‐wave rectified EMG antialiased data. Two measures of EMG amplitude were extracted: first the area under the curve was determined for the first 200 ms and second, a low‐pass filter (6 Hz) was applied to the full‐wave rectified data in an effort to smooth data to extract absolute EMG peak amplitude. Both of these amplitudes were obtained from each epoch and then averaged for each participant for each time condition. In addition, time between each movement, termed the inter movement interval, was extracted from the continuous dataset for each participant, for each time condition, and then averaged.

### Statistical analysis

All data were statistically analyzed using one way repeated measures ANOVA with time relative to exercise as the factor. Since experiments 1 and 2 only differed by removal of self‐monitoring cycle cadence and 2 additional time measures in experiment 2, a mixed two‐way ANOVA was conducted with the first three time measures (Pre, Post, Post(Rest)) with time and exercise condition (self‐monitoring vs. fixed cadence) as factors. Planned contrasts to test the hypothesis of the transient nature of the aerobic exercise modulation on early BP were completed. Post hoc analyses were performed with the Tukey correction method to investigate differences between time points for any of the other specific components. A t‐test was used to compare the difference of actual to target exercise heart rate between the two experiments.

## Results

### Experiment 1

#### Bereitschaftspotential

All subjects (*n* = 11) presented with a similar BP morphology prior to movement onset. After elimination of contaminated epochs, approximately 100 epochs per subject per condition were included in the analysis. The greatest absolute BP peak was maximal over the vertex (Cz) for all time points (Cz Mean ± standard error: Pre; −8.59 *μ*V ± 3.38, Post; −9.31 *μ*V ± 2.15, Post(Rest); −13.38 *μ*V ± 4.63). There was no main effect of exercise over time on either absolute peak amplitude (*P* = 0.779) or peak latency (*P* = 0.201).

**Figure 3.**
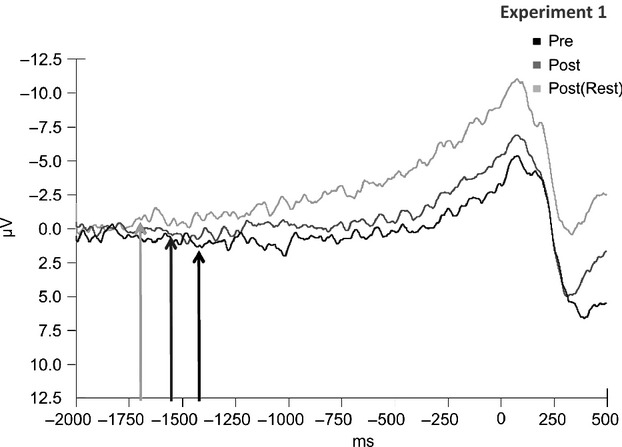
Grand average BP tracings (*n* = 11, ~100 epochs/subject) prior to movement onset (t = 0) of Pre, Post, and Post(Rest) time conditions following aerobic exercise at the Cz electrode. Data are baseline corrected (−2000 to −1800), band pass filtered (0.1–30 Hz), and arrows indicate group average early BP onsets (Pre: −1448 ms, Post: −1563 ms, Post(Rest): −1696 ms).

There was a significant effect of time relative to exercise on the early BP onset latency at the Cz electrode (*F*_2,10_ = 8.658, *P* = 0.002). However, planned contrasts revealed significant differences in the early BP onset only between Pre and Post(Rest) (*F*_1,10_ = 17.062, *P* = 0.002) and not between Pre and Post conditions (*F*_1,10_ = 2.972, *P* = 0.115), depicted in Figure 3. Further analyses regarding late BP slope and amplitude of the RAP revealed no significant main effect of time relative to exercise (*P* = 0.666 and 0.178, respectively).

#### Electromyography

The EMG data revealed no significant differences across time conditions for the ECR absolute peak amplitude (*P* = 0.113), suggesting that ECR activity was similar across the time conditions. Furthermore, intermovement intervals were not significantly altered across differing time conditions (*P* = 0.770), indicative that participants were able to maintain similar pace during each testing block. Finally an analysis of the ballistic nature of movement revealed nonsignificant differences between time conditions for the area under the curve of the first 200 ms of EMG activity (*P* = 0.701). Overall these data suggest that participants did not alter their movement behavior between trials.

### Experiment 2

#### Bereitschaftspotential

**Figure 4.**
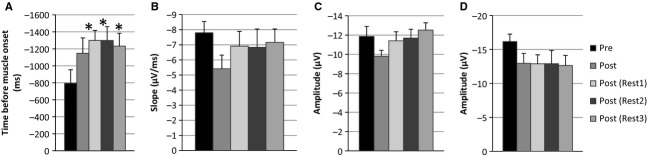
(A) Early BP onset (*n* = 8) represented as time (seconds) prior to EMG initiation. *represents significant difference (*P* < 0.05) as compared to Pre. (B) Late BP slope (*n* = 10) in *μ*V/ms revealed no significant differences across time (*P* > 0.05). (C) Motor potential amplitude (*n* = 10) expressed no significant differences across time. Finally (D) reafferent potential peak to peak amplitude (*n* = 10) difference revealed a main effect of time (*P* < 0.05) but post hoc Tukey revealed no differences between Pre versus Post, Post(Rest1), Post(Rest2), and Post(Rest3) (*P* > 0.05).

All subjects included in analysis (*n* = 11) presented with similar BP morphology occurring prior to EMG onset, except three individuals who presented with no early BP while all other BP characteristics including late and reafferent BP components remained intact. For the purpose of analysis these three subjects were removed from early BP onset evaluation but remain in late BP analysis. Following postanalysis blink removal, an average of 120 epochs per subject per time condition contributed to individual averages. Motor potential was maximal over central electrode sites (Fig. 4).

A one way repeated measures ANOVA revealed a main effect of time following aerobic exercise on early BP (*F*_4,7_ = 3.370, *P* = 0.023), appeared as alterations in earlier onsets. A priori contrasts confirmed results from the first experiment in that no changes occurred immediately after exercise cessation (Post) compared to the pre‐condition (*F*_1,7_ = 2.899, *P* = 0.132). However, significant differences were observed when heart rate returned to baseline at Post(Rest1) (*F*_1,7_ = 46.597, *P* = 0.00001). Post hoc Tukey test revealed this effect was sustained for up to an additional 15 min in Post(Rest2) (*P* = 0.0152) with the effect not reaching significance at the Post(Rest3) (*P* = 0.1318) time condition.

In further analysis of experiment 2 data, there were no significant differences over time (Pre to Post(Rest3) in BP slope (*P* = 0.290), motor potential amplitude (*P* = 0.884) or latency (*P* = 0.351) at the Cz electrode site. However, there was a significant main effect of time on RAP amplitude (*F*_4,9_ = 2.722, *P* = 0.045) but not on latency (*P* = 0.615). Tukey's test revealed no significant differences (*P* > 0.05) on motor evoked sensory information between RAP amplitude sustained over all post exercise time measures compared to Pre.

**Figure 5.**
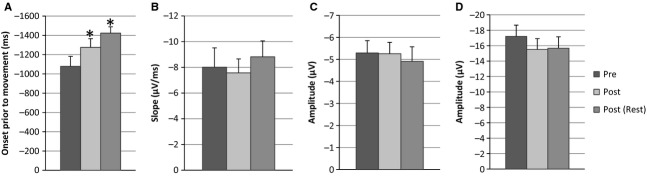
(A) Early BP onset (*n* = 20) represented as time (ms) prior to EMG initiation. Significant differences as depicted by *between Pre versus Post (*P* < 0.05) and Pre versus Post(Rest) (*P* < 0.01). (B) Late BP slope (*n* = 22) in *μ*V/ms revealed no significant differences across time (*P* > 0.05). (C) Motor potential amplitude (*n* = 22) expressed no significant differences across time. Finally (D) reafferent potential peak to peak amplitude (*n* = 22) difference revealed nonsignificant results across time (*P* > 0.05).

A mixed two‐way ANOVA, with time and exercise condition as factors, confirmed no significant differences between experiment 1 and 2 exercising protocols (*P* = 0.442). In addition exercise heart rates between both experiments were analyzed as an absolute difference in subject target exercise and exercise actual heart rate with no significant differences between exercise 1 (7.29 ± 2.19 bpm) and exercise 2 (6.28 ± 1.41 bpm) (*t*_21_ = 0.332, *P* = 0.74). The nondifference in exercising heart rate between experiments is indicative that the different exercise protocols yielded a similar exercise dose. When early BP data between experiment 1 and 2 were combined for time points up to Post(Rest1), a main effect of exercise over time occurred. Differences occurred both immediately after exercise (Post condition) (*F*_1,19_ = 5.245, *P* = 0.034) and at the Post(Rest1) condition (*F*_1,19_ = 45.214, *P* = 0.0001) (Fig. 5).

Further combined analysis affirmed experiment 1 results with no observed main effect of time on late BP slope (*P* = 0.676), motor potential amplitude (*P* = 0.738), or RAP amplitude (*P* = 0.430) at the Cz electrode site.

#### Short latency somatosensory evoked potentials

All participants (*n* = 11) had a positive P20 and negative N30 peaking over fronto‐central (FCz, and Cz) electrode sites. A one way repeated measures ANOVA revealed no main effect of time in absolute peak N30 amplitude at Cz (*P* = 0.252) or FCz (*P* = 0.359) electrode sites. Further peak to peak analysis between N30 and P20 presented no main effect of time on N30 amplitude at FCz (*P* = 0.293) or Cz (*P* = 0.092) following aerobic exercise.

#### Electromyography

There were no significant differences between conditions for the ECR absolute peak amplitude (*P* = 0.112; Mean: Pre = 0.303 mV ± 0.03, Post = 0.236 mV ± 0.03, Post(Rest1) = 0.231 mV ± 0.02, Post(Rest2) = 0.243 ± 0.03, Post(Rest3) = 0.235 ± 0.03). Furthermore, intermovement intervals were not significantly altered across differing time conditions (*P* = 0.123; Mean: Pre = 4.15 sec ± 0.35, Post = 3.85 sec ± 0.36, Post(Rest) = 4.31 sec ± 0.29, Post(Rest2) = 4.10 sec ± 0.28, Post(Rest3) = 3.93 sec ±0.28). Finally, there were no significant differences between time conditions for the area under the curve of the first 200 ms of EMG activity (*P* = 0.091; Mean: Pre = 35.89 ±3.02, Post = 26.25 ± 2.71, Post(Rest) = 25.39 ± 3.00, Post(Rest2) = 26.01 ± 4.43, Post(Rest3) = 26.0794 ± 5.12). These data suggest, like experiment 1, that EMG and movement characteristics were similar across different times relative to exercise.

## Discussion

To our knowledge, the current study is the first to show that a 20 min bout of aerobic exercise at a moderate intensity (70% age‐predicted maximum heart rate) on a recumbent cycle ergometer alters early motor cortical processing. In support of our initial hypothesis, an acute bout of aerobic exercise was sufficient to elicit a change in cortical activity during a motor task immediately after aerobic exercise that remained elevated for roughly an hour following exercise cessation.

Our results are in line with previous findings evaluating acute aerobic exercise and other EEG recordings such as the CNV (Kamijo et al. 2004a), frontal asymmetry (Petruzzello and Tate 1997) and P300 (Hillman et al. 2003; Kamijo et al. 2004b, 2009; O'Leary et al. 2011), which found changes in ERP immediately following aerobic exercise. However, our investigations regarding the temporal nature of this effect on EEG recordings are less common. To our knowledge only one other investigation by Joyce et al. (2009) revealed beneficial effects of an acute bout of moderate aerobic exercise on response execution and inhibition sustained for up to 52 min after exercise cessation. The temporality of our results was more closely related with this study in that the EEG changes lasted at least an hour following exercise cessation.

The neural mechanisms driving the cortical changes postexercise are still unclear. Recent meta‐analytical models have attempted to provide a foundation for neural mechanisms behind the influence aerobic exercise has on cognitive markers (McMorris and Hale 2012). An emerging model known as the Reticular Activating Hypofrontality (RAH) model, developed by Dietrich and Audiffren (Dietrich and Audiffren 2011) aims to explain the combination and synergistic role aerobic exercise has on enhancing arousal and decreasing conflicting cognitive pathways. Aerobic exercise by nature increases the cognitive load on the CNS requiring a reallocation of resources for implicit information processing. Due to this, higher order functions of the prefrontal cortex disengage not only as a result of resource limitation but in an effort to minimize the conflict they may have on implicit processing. However, one major limitation to this model is that it is designed to explain the effects during or immediately following aerobic exercise and not sometime after exercise cessation. The current study utilized heart rate as a surrogate for CNS arousal. Resting heart rate fluctuations have been shown to be related to changes in functional connectivity within cortical and subcortical structures that have previously been shown to mediate arousal (Chang et al. 2014). Generally speaking, exercise enhances the sympathetic nervous system output (sympathetic drive) in an effort to maintain the demands of the exercise (Christensen and Galbo 1983). This alteration in sympathetic drive manifests peripherally as changes in HR and centrally as changes in CNS arousal (Porges 2003). Further support suggests that aerobic exercise has a direct influence on the reticular activating process by direct connections between brainstem areas that control heart rate and respiration (Fowles 1980; Porges 2003). In the current study, we used increasing heart rate as a measure of increased sympathetic drive in an effort to indirectly measure central arousal following aerobic exercise and found that cortical changes lasted long after heart rate returned to baseline. Therefore, it is possible that arousal may be contributing to the immediate modulations in early BP morphology but cannot be considered the sole contributor to this effect as it is inadequate to explain the temporal nature of these adaptations.

A more reasonable substitute to explain current BP adaptations (selective to early BP) may be that particular cortical networks respond more favorably to exercise. Previous work has shown that both early and late BP components are differentially sensitive to pharmacological manipulation, where acute administration of dopaminergic medication enhances early BP morphology with no change to late BP (Dick et al. 1987). Further research involving healthy animal models lends additional support for this hypothesis; showing increases in dopamine and neurotransmitter synthesis and release within basal ganglia networks during an acute bout of exercise (Kindermann et al. 1982; Chaouloff 1989; Meeusen and De Meirleir 1995), reaching maximal concentration 40–60 min into recovery (Hattori et al. 1994). Taken together these studies may suggest that exercise, as is the case with pharmacological manipulation, selectively alters the neurotransmission within the basal ganglia – SMA network manifesting as enhanced cortical excitability depicted in morphological changes in early BP.

### Bereitschaftspotential

Results from both experiment 1 and 2 suggest that early and not late component BP is influenced by a moderately intense acute bout of aerobic exercise. Upon inspection of the combined means the exercise‐induced influence on early BP appears to be a graded response, rising immediately after exercise, peaking when heart rate returns to baseline, and slowly degrading. Interestingly, the effect of the exercise appears to be selective in altering only the early component, with no significant modulations developing in late BP slope, motor potential or RAP. In contrast, previous reports have shown that early BP can be selectively modulated when level of intention, preparatory state, force and selection of movement, and when learning paradigms are employed (Shibasaki and Hallett 2006). As the current task did not involve a learning paradigm or changes in movement characteristics, a more reasonable explanation for these changes may be that exercise alters the participant's preparatory state or intent to move. One method previously employed to test preparatory state has been to use a split attention task, where participants are to perform a cognitive task following each movement (VaezMousavi and Barry 1993). VaezMousavi and Barry's (1993) findings supported a possible role for attentional resources during early BP development. As previous studies using exercise have been shown to enhance P300 and CNV amplitude (scalp recorded potentials reflective of attentional resources), it seems logical that a similar mechanism could explain the current findings. To the contrary our study is the first to show a main effect of time on RAP amplitude following aerobic exercise, even though this effect only occurred in experiment 2. However, Tukey's post hoc analysis revealed no significant differences between pre and any post time measure. Since RAP is thought to represent reafferent limb information following movement production (Bötzel et al. 1997), these findings may provide evidence for possible modulation within primary somatosensory cortices. The current data are insufficient to clearly decipher the role aerobic exercise has on S1 and therefore any attempt to draw conclusions from these data would be merely speculative. That being said the current study warrants further investigation of the role aerobic exercise may have on modulating the somatosensory system.

### Short latency SEP

In addition to the BP in study 2, the short latency somatosensory evoked potential, the N30 was analyzed. Based on results from experiment 1 that early but not late BP was altered, it was hypothesized that another scalp recorded potential with similar cortical generators would provide further insights. The cortical generator of the N30 component still remains subject to debate, although the SMA likely contributes to it (Barba et al. 2005; Legon et al. 2013). What is agreed upon is that the N30 is clearly influenced by motor behavior (Legon et al. 2010), and is influenced in some pathological states similar to that of the BP (Gironell et al. 2002). Based on the assumption that the N30 and BP share similar generators, we would expect to see a modulation of N30 amplitude similar to that of the BP. Contrary to the original hypothesis; the current data suggest that an acute session of aerobic exercise may influence incoming movement afferent information differently than outgoing motor information. Although the modulations to N30 did not reach significance, further investigations should examine the possible effect of aerobic exercise on somatosensory evoked potentials.

### Strengths & limitations

It is well accepted within the literature that BP measures are sensitive to changes in movement profiles (See review Shibasaki and Hallett 2006). In the current study subjects were requested to produce a ballistic wrist extension of similar magnitude and direction. Our profiling of EMG amplitude, pace, and ballistic nature in all subjects reported no significant differences over time through the entirety of the testing blocks. This supports that changes seen to early BP are not related to alterations in movement characteristics between the separated measurements.

In addition to the fundamental process underlying the movement, characteristics of the sample population must be considered. Emerging evidence supports the role for participant fitness in altering basal levels of neuroelectric indices (Kamijo et al. 2010). Based on the data gained from the IPAQ scores, it was determined that our participants were classified as highly active (with the exception of two participants) which in turn could relate to their individual fitness. Although the influence fitness has on BP morphology was not of primary hypothesis in this study, these data may suggest a future research direction.

Finally, due to the limitations that the BP is detected using scalp recordings, it is possible that the BP was not sensitive enough to determine subtle changes in motor execution areas such as M1. However, some reports using transcranial magnetic stimulation, a more sensitive marker of motor corticospinal output, have suggested modulations within M1 following acute aerobic exercise (Yamaguchi et al. 2012; Singh et al. 2014; Smith et al. 2014).

### Future considerations

With regard to premovement BP morphology, previous reports in PD show the early BP to be greatly attenuated or nonexistent while the later BP component remains unchanged or is larger than normal (Dick et al. 1989). However, to date, no known study exists using aerobic exercise, PD, and the BP. Recent support has shown that, in combination with regular PD medication (Bergen et al. 2002) and when medication is removed (Müller and Muhlack 2010), aerobic exercise not only improves cardiovascular function but also alleviates common symptoms of akinesia and bradykinesia when compared to age‐ and behavior‐matched controls. One interesting venue for exploration is whether aerobic exercise is able to recapture early BP in PD patients.

## Conclusion

An acute bout of moderately intense aerobic exercise was associated with selective changes to early motor‐related cortical output. These effects lasted over an hour after the cessation of exercise. This study provides further support that aerobic exercise influences not only the cognitive but the motor preparatory components of movement. These results although conducted in healthy controls, may provide a further avenue of exploration in patient populations such as those suffering from PD.

## Conflict of Interest

None declared.
